# Western lifestyle linked to maladaptive trained immunity

**DOI:** 10.7554/eLife.105835

**Published:** 2026-01-21

**Authors:** Aurelia Josephine Merbecks, Christabel Mennicken, Dennis Marinus de Graaf, Kateryna Shkarina, Theresa Wagner, Eicke Latz

**Affiliations:** 1 Institute of Innate Immunity, University Hospital Bonn Bonn Germany; 2 Deutsches Rheuma-Forschungszentrum (DRFZ) Berlin Germany; Radboud University Medical Center Netherlands; National Institute of Immunology India

**Keywords:** trained immunity, maladaptive, autoinflammation, infectious disease, Western diet, interleukin-1

## Abstract

Trained immunity (TI) refers to a state of innate immune cells that, after encountering an initial stimulus and undergoing epigenetic reprogramming and metabolic changes, allows them to respond more effectively to a subsequent challenge. TI yields a survival advantage, particularly in a pathogen-rich context. However, maladaptive TI may damage the host by exacerbating inflammatory diseases. Here we review which aspects of Western lifestyle may contribute to maladaptive TI, including a Western diet, periodontitis, chronic psychological stress, and environmental triggers such as air pollution and microplastics. Finally, we consider lifestyle intervention as a way to prevent or reduce the impact of maladaptive TI.

## Introduction

Immune responses need to be dynamic and capable of adjusting to the context in which immune stimuli are encountered. The cellular responses towards stimuli include classical activation, priming, tolerance, and trained immunity (TI) (Netea and Joosten, 2024). Upon classical activation, the antimicrobial capacity of immune cells is used to resolve an immediate threat. Priming temporarily enhances the cells’ ability to manage future challenges, leading to an additive or synergistic effect upon a secondary challenge, often achieved via the upregulation of surface or intracellular receptors. Tolerance means that a cell becomes less responsive or even unable to respond to a subsequent stimulation. In contrast to the previous three states which describe an immediate or short-term cellular response, TI refers to a state of ‘memory’ that exists after a primary stimulation induces reversible epigenetic and metabolic reprogramming. Once trained, a cell returns to a basal level of functioning (homeostasis), ready to launch an amplified or prolonged inflammatory response upon exposure to a subsequent stimulus (Netea et al., 2016). When cells are exposed to stimuli for a prolonged period, the distinction between priming and training may become blurred. Under these conditions, cells may enter a hybrid state, that is, carrying both traits of TI and traits of prolonged priming. This overlap is particularly difficult to assess when positive feedback loops are involved, such as those driving IL-1β production in autoinflammatory diseases. Careful consideration should be given to the stimulus, its dosing and clearance, as well as the timepoint of assessing the inflammatory response, as each of these may influence the extent to which priming and TI influence the response to a secondary stimulus.

TI allows plants and invertebrate animals to build an immune memory that can be recalled upon subsequent (unrelated) infections or other forms of insults. In contrast, the long-lasting memory of the adaptive immune system is present only in jawed vertebrates, which represent approximately 3% of living species on Earth (Melillo et al., 2018). TI yields an evolutionary advantage by providing non-specific protection against infection, which is particularly beneficial when the host is under threat of multiple pathogens or lacks specific immune memory. The programme of TI remains present even in species that have evolved an adaptive immune system, suggesting an evolutionary pressure that maintains this form of memory, or at least that retaining TI capacity is a tolerable burden. TI can be induced by certain pathogen-associated molecular patterns (PAMPs) and danger-associated molecular patterns, which can arise both during infection and associated tissue damage, or as a consequence of lifestyle or environmental exposure. Since trainable cells cannot distinguish between a beneficial or a maladaptive training, primary or secondary stimulus and whether its response is beneficial or detrimental to the host, maladaptive TI can occur upon aberrant stimulation (e.g., the consumption of an inflammatory, Western diet), the lack of target specificity (e.g., increased myelopoiesis or inflammatory cytokine production), as well as the failure to timely resolve or limit an immune response. Maladaptive TI contributes to pathological inflammation and/or the development of chronic inflammatory diseases (Fanucchi et al., 2021; Netea et al., 2020; Ochando et al., 2023); such as atherosclerosis (Dong et al., 2024), cardiac dysfunction (Simats et al., 2024), arthritis (Li et al., 2022), and multimorbidity as seen in cardiac dysfunction, as well as skeletal muscle and kidney vulnerability (Nakayama et al., 2024; Ochando et al., 2023). The prevalence of these non-communicable diseases (NCDs) increases with age and concurrently, TI shifts from being solely a protective mechanism to also becoming detrimental to the host. However, the protective effect against infections TI offers in early life enhances an individual’s reproductive fitness. Thus, maladaptive TI fits the concept of antagonistic pleiotropy, where the survival benefits that TI provides in early life outweigh the detrimental effects in later life. In the last century, maladaptive TI has become a more persistent burden on our ageing society. This review aims to place maladaptive TI in the context of NCDs and modern lifestyle, involving industrialisation, pollution, and Western diets. We focus on risk factors for NCDs, which may also be risk factors for maladaptive TI.

Box 1. **Key messages**

- Trained immunity can protect against infectious diseases
- Maladaptive trained immunity may exacerbate non-communicable diseases
- Aspects of Western lifestyle can trigger maladaptive trained immunity
- These aspects include a sedentary lifestyle, inflammatory diet, a polluted environment and psychological stress
- Lifestyle interventions may mitigate maladaptive trained immunity and non-communicable diseases

## Basic mechanisms of TI

In 2012, Kleinnijenhuis et al., 2012 first demonstrated the existence of TI through the nucleotide-binding oligomerisation domain-containing protein 2 (NOD2)-dependent non-specific protective effects of the Bacillus Calmette–Guérin vaccine against reinfections. Since then, the list of known TI inducers has grown. The core mechanism of TI is consistent: intracellular signalling cascades, often including the activation of the mTOR–HIF-1α axis, drive metabolic reprogramming and the induction of epigenetic modifications that are sustained even after the initial stimulus is cleared, enabling a rapid and heightened inflammatory response upon a secondary stimulus.

Metabolic reprogramming involves increased oxidative phosphorylation, aerobic glycolysis, TCA cycle, glutaminolysis, and fatty acid metabolism (Ferreira et al., 2024). This ultimately leads to enhanced energy production in the form of adenosine triphosphate and nicotinamide adenine dinucleotide, and the accumulation of signalling metabolites in the cell, for example, fumarate, succinate, and malate, which confer immunometabolic and epigenetic changes (Arts et al., 2016a, Ferreira et al., 2024). For example, glutaminolysis and cholesterol synthesis, in combination with the elevated glycolysis, mediate epigenetic changes via trimethylation of histone H3 at lysine 4 (H3K4me3) and histone H3 lysine 27 acetylation (H3K27ac) which change chromatin accessibility of metabolic pathways (Arts et al., 2016b; Xu et al., 2023). The enzyme Set7 catalyses the methylation of H3K4me, leading to transcriptional activation and thus plays an important role in β-glucan-induced TI (Keating et al., 2020; Gates et al., 2024). For a more detailed overview of the metabolic regulation and epigenetic rewiring during TI induction, we refer to recent reviews (Ferreira et al., 2024).

## Peripheral TI

It is important to distinguish between peripheral and central TI. Peripheral TI in mature immune cell populations is typically non-inheritable, important for rapid defence in specific tissues but with a transient impact. However, peripheral TI may also have long-lasting effects in long-lived cell populations with self-renewing capacity, such as alveolar macrophages (Yao et al., 2018) and microglia (Rossi and Lewis, 2018). To date, the phenomenon of peripheral TI inflammatory stimuli has been described in multiple cell types, including monocytes and tissue-resident macrophages (Netea et al., 2016), neutrophils (Moorlag et al., 2020), NK cells (Kleinnijenhuis et al., 2014; Schlums et al., 2015), dendritic cells (Hole et al., 2019), and innate lymphoid cells (Serafini et al., 2022) as well as many non-immune cell types (Hamada et al., 2018).

## Central TI in the bone marrow

In contrast to peripheral TI, central TI is typically sustained over longer periods of time (Ochando et al., 2023; Johansson et al., 2023) and is characterised by persistent immunometabolic, epigenetic, and transcriptional changes in haematopoietic stem and progenitor cells (HSPCs) (Mitroulis et al., 2018; Kaufmann et al., 2018; Christ et al., 2018). Central TI is established in the bone marrow, where HSPCs are maintained, self-renew and differentiate, thereby regulating haematopoiesis and egression of cells into the circulation (Morrison and Scadden, 2014; Mendelson and Frenette, 2014). The bone marrow is highly dynamic and adapts to inflammatory stimuli including infections (Burberry et al., 2014; Manz and Boettcher, 2014; Takizawa et al., 2011), ageing (Mitchell et al., 2023; Woods and Guezguez, 2021; Ambrosi et al., 2017), and metabolic imbalances (Ambrosi et al., 2017; Singer et al., 2014). One example of such adaptation is emergency myelopoiesis, which can be triggered by infection, inflammation or myeloablative insult such as chemotherapy (Swann et al., 2024; Chavakis et al., 2019; Manz and Boettcher, 2014). It can also be induced by cytokines, growth factors, or PAMPs (Demel et al., 2022). These stimuli enhance differentiation of myeloid-biased haematopoietic stem cell (HSC) populations and increase proliferation of lineage-committed granulocyte–monocyte progenitors (GMPs).

The long-term effects of central TI on HSCs have been elegantly demonstrated in transplantation studies (Mitroulis et al., 2018). Mice receiving long-term HSCs (LT-HSCs) from β-glucan-trained mice exhibited a higher proportion of myeloid cells and a reduction in B cells. In addition to IL-1β, IFNs, for example, IFNα are also crucial in the induction of TI by β-glucan (Kalafati et al., 2020), and reprogram bone marrow GMPs, resulting in neutrophils with enhanced anti-tumour immunity. In mice, intraperitoneal injection of β-glucan results in the upregulation of IL-1β and GM-CSF, activating the IL-1R1 and GM-CSF receptors, promoting the expansion of myeloid-skewed HSPCs in the bone marrow, including myeloid-biased CD41+ LT-HSCs (Mitroulis et al., 2018), following metabolic rewiring. TI may thus lower the threshold for emergency haematopoiesis.

Beneficial effects of central TI are expected in the context of microbial and viral infections and potentially cancer, as an elevated immune response supports clearance of the pathogen and protection against reinfection or killing of tumour cells, respectively. However, maladaptive central TI may exacerbate inflammatory NCDs.

In chronic inflammatory diseases, a combination of TI, (chronic or) emergency haematopoiesis and priming due to unresolved inflammation may each contribute to the number of innate immune cells and an inflammatory phenotype. Notably, persistent emergency haematopoiesis and central TI have significant mechanistic overlap, yet different outcomes (Swann et al., 2024). For example, inflammatory ligands can drive myeloid-skewed differentiation of HSPCs as part of emergency myelopoiesis, increasing myeloid cell output without necessarily inducing memory-like traits in innate immune cells. Moreover, alterations of the local bone marrow environment may enhance myelopoiesis without conferring immune memory. In contrast, prior LPS exposure can reprogramme HSCs epigenetically, boosting their response to Gram-negative bacteria and improving clearance (de Laval et al., 2020).

## Pro-inflammatory memory in non-immune cells

While the main focus of the TI field has so far been on the innate immune system, it is increasingly recognised that certain non-immune cell subsets can also undergo long-term epigenetic and functional reprogramming in response to pro-inflammatory cues, termed as ‘pro-inflammatory memory’ (Naik and Fuchs, 2022; Larsen et al., 2021). To date, such memory has been described in various epithelial and non-epithelial cell types, fibroblasts (Cheng et al., 2023) and smooth muscle cells (Villeneuve et al., 2008). Following local inflammation or wounding, this type of memory has been shown to be mostly restricted to or around the primarily affected site (Naik et al., 2017; Levra Levron et al., 2023). In contrast, systemic stimuli, such as infection or metabolic dysregulation, result in training in multiple tissues and organs and can even induce reprogramming in the offspring if encountered during pregnancy (Nash et al., 2023; Lim et al., 2021).

Although beneficial for enhancing tissue repair or resistance to infections, non-immune memory can also have maladaptive consequences. In mouse models, pre-existing inflammatory memory in skin and pancreas (Naik et al., 2017; Falvo et al., 2023) accelerated tissue healing but lowered the threshold for neoplastic transformation and tumour development (Greten and Grivennikov, 2019). Post-inflammatory loss of epithelial diversity and epithelial dysfunction has been observed in the mouse model of acute gastrointestinal graft-versus-host disease, where it manifested as metabolic defects and therefore, reduced regenerative and differentiation capacity of intestinal stem cells (Zhao et al., 2024). Finally, in diabetic mouse models and in vitro hyperglycaemia-exposed endothelial cells, inflammatory reprogramming promoted pro-inflammatory cytokine production, as well as monocyte adhesion and transmigration, contributing to vascular inflammation (Sohrabi et al., 2020; Brasacchio et al., 2009; Villeneuve et al., 2008).

## Potential triggers of maladaptive TI

Multiple kinds of TI triggers exist, including lifestyle factors and environmental exposure, as well as several other factors, which are beyond the scope of this review including infections, genetic predisposition, and sterile injury. Many of these triggers are themselves risk factors for chronic inflammatory disease, which exacerbate chronic inflammation and contribute to disease progression by mechanisms including, but not limited to, TI. We will discuss different aspects of a Western lifestyle, including environmental and behavioural factors, that may induce maladaptive TI.

### Urbanisation and pollutants

Dysregulation of the immune system in response to diet and urbanisation is well studied, such as by Temba et al., 2021. The authors compared the immune states of rural and urban Tanzanians and reported elevated inflammatory signalling in the urban population, with elevated transcription in pro-inflammatory cytokines including interferons. The rural population, in contrast, presented with higher levels of the anti-inflammatory metabolites apigenin, trehalose and itaconate in the circulation, mainly attributed to differences in diet (Temba et al., 2021). A follow-up study provided further insights into Western diet-induced maladaptive TI. Switching to a Western diet, for as little as four weeks, led to persistent immunometabolic changes associated with NCDs and induced highly pro-inflammatory states. Notably, these alterations included increased neutrophil counts and activation, elevated inflammatory and cardiometabolic proteins, and sustained transcriptional changes in immune-related genes (Temba et al., 2025). With regards to food intake, epidemiological studies from multiple countries demonstrate that the prenatal exposure to highly disruptive events, such as famines, is associated with the increased risk of type 2 diabetes and other TI-related metabolic disorders (Wang et al., 2020; Lumey et al., 2024; Ekamper et al., 2014).

Additional environmental factors that have a great impact on human health include micro- and nanoplastics (MNPs). MNPs can accumulate in the body and trigger immune responses, for instance in human peripheral blood mononuclear cells (PBMCs) (Han et al., 2020) and murine intestines (Li et al., 2020). The abundance of MNPs in urban areas is much higher than in rural areas (Liao et al., 2021; Liu et al., 2019). MNP uptake and burden also depend on the food intake and food packaging (Feng et al., 2023). Furthermore, circulating Bisphenol-A (BPA) is positively correlated to TNF in the serum of healthy individuals. BPA was recently proposed as a new TI inducer (Dallio et al., 2023), although the training effects caused by MNPs and BPA need to be further investigated.

Nanoparticles, such as pristine graphene, and metal particles from air pollution, adjuvants, drug carriers, and prosthetics, can also influence the immune system (Munoz-Wolf and Lavelle, 2021). While pristine graphene particles do not cause an immune reaction themselves, murine bone marrow-derived macrophages (BMDMs) stimulated with TLR ligands after being exposed to pristine graphene particles show an enhanced secretion of TNF and IL-6 while having reduced IL-10 levels (Lebre et al., 2020). Quantum dots have also been implicated in altering cellular metabolism, resulting in lipid accumulation in vitro in mouse and rat cell lines. This was induced in an HIF-1α-dependent manner and accompanied by downregulation of fatty acid oxidation (Przybytkowski et al., 2009). Miller et al., 2024 reported that high levels of fine particulate matter in the air are associated with high inflammatory responses to ex vivo stimulated whole blood of children. Movassagh et al., 2023 also showed that fine particulate matter induced TI in circulating monocytes, resulting in the heightened inflammatory state and accumulation of H3K27ac marks. Maladaptive TI could also explain the role of fine particulate matter as air pollutant in worsening of COVID-19 infections (Kerboua, 2020).

Overall, these results suggest that polluted environments may cause TI and a dysregulated inflammatory phenotype. Taking care of our environment therefore becomes an even more critical target to reach a healthier and sustainable future, especially in light of the expected challenges with regard to climate change (Else and Cruickshank, 2025).

### Chronic psychological stress

Prolonged or chronic psychological stress (CPS) increases the risk of life-threatening infections as well as NCDs such as cardiovascular, metabolic, neurodegenerative, and autoimmune diseases or cancer (Song et al., 2018; Song et al., 2019; Song et al., 2020; Vignjević Petrinović et al., 2023). Both in humans and in animal models, CPS was shown to be associated with enhanced local and systemic inflammation. Elevated stress is often accompanied by increased systemic- and cerebrospinal levels of pro-inflammatory cytokines, such as IL-1β, IL-6, and TNF (Levine et al., 1999; Golovatscka et al., 2012; Miller et al., 2009), as well as significant transcriptional and functional alterations in circulating innate immune cells, characterised by upregulation of pro-inflammatory genes (Schneider et al., 2023; Powell et al., 2013; Cole et al., 2015; Cole et al., 2012). Subsequent studies in mice revealed stress-induced increases in the proliferation of HSCs (Heidt et al., 2014), as well as their mobilisation from bone marrow into the circulation and establishment of secondary haematopoietic sites in the spleen (McKim et al., 2018), which together mediate the increased production of the pro-inflammatory monocytes and neutrophils in stressed animals. Finally, monocytes isolated from stressed humans and mice display a characteristic immunometabolic and epigenetic rewiring and inflammatory hyperresponsiveness reminiscent of those induced by more typical TI inducers, such as β-glucan (Barrett et al., 2021).

While the exact molecular mechanisms of CPS-induced TI remain to be fully defined, it is known CPS activates the sympathetic nervous system and hypothalamic-pituitary-adrenal (HPA) axis, both of which are known to have pleiotropic effects on the immune system. The stress hormone catecholamine has been proposed to directly act as training stimuli. Catecholamine induced β-adrenoreceptor-mediated epigenetic and transcriptional reprogramming of isolated human monocytes. Patients with elevated catecholamine levels also displayed typical training hallmarks (van der Heijden et al., 2020).

### Parental diet may drive TI in offspring

Parental diet or a high parental BMI may also lead to innate inflammatory memory in offspring and lead to metabolic disorders (e.g., diabetes), particularly in male offspring. This may be driven by mitochondrial transfer RNA (mt-tRNA) present in the sperm, which prematurely activates oxidative metabolism. In mice, a high-fat diet induces stress in paternal mitochondria within spermatozoa and increased production of mt-tRNA and their fragments (Tomar et al., 2024). The functional role of these fragments was assessed by injecting sperm RNA from high-fat diet and normal diet fed mice into normal zygotes, resulting in metabolic disturbances in the offspring (Holoch and Moazed, 2015) highlighting that small non-coding RNA, such as tRNA fragments, can induce epigenetic changes. Yet, Chen et al., 2016 observed that despite glucose intolerance and shifts in gene expression profiles, these were not associated with changes in gene methylation patterns. Instead, many of the tRNA fragments aligned with promoter regions, suggesting a potential regulatory mechanism, which needs to be further elucidated. The same phenomenon has been observed in humans, where a high paternal BMI at the time of conception imprinted as metabolic malfunctioning in the offspring. Notably, the amount of small mt-tRNA fragments in sperm correlates positively with BMI (Tomar et al., 2024; Chen et al., 2016). Maternal high-fat diet has been shown to prime the neonatal immune system and contribute to paediatric non-alcoholic fatty liver disease (Jonscher et al., 2020). Recently, maternal obesity has been implicated with reprogramming fetal Kupffer cells, resulting in a pro-inflammatory and HIF-1α-dependent metabolic reprogramming, ultimately driving fatty liver disease in offspring (Huang et al., 2025).

### Periodontitis

Periodontitis is considered a lifestyle disease and is an example of central maladaptive TI. Neglected periodontitis can give rise to tooth-biofilm dysbiosis, ulcer formation, and bleeding. Exposed (micro)vasculature enables translocation of periodontal bacteria that causes local inflammatory responses, with the potential of spill-over into the circulation and progress to systemic bacteraemia, leading to chronic inflammation (Vitkov et al., 2021).

Li et al., 2020 performed ligature-induced periodontitis (LIP) in mice and reported an IL-1-dependent central TI phenotype, including bias towards myelopoiesis. Strikingly, upon transfer of bone marrow from LIP mice to naive mice, an exacerbated collagen antibody-induced arthritis (CAIA) phenotype was observed. Similarly, transplanted bone marrow from mice subjected to CAIA to naive mice gave rise to an exacerbated LIP. Crucially, mice deficient for *Il1r1* in only the HSPC compartment were not readily trained. Potentially connected via central TI, periodontitis in patients has been associated with rheumatoid arthritis (Bartold and Lopez-Oliva, 2020), psoriasis (Nijakowski et al., 2022), inflammatory bowel disease (Newman and Kamada, 2022), diabetes mellitus (Preshaw et al., 2012), Alzheimer’s disease (Dioguardi et al., 2020), non-alcoholic fatty liver disease (Hatasa et al., 2021), and cardiovascular disease (Sanz et al., 2020). Long-term clinical trials focused on improving oral health to reduce periodontitis and incidence of NCDs as an end-point would confirm this mechanism in humans.

### Cardiometabolic diseases

Dietary habits influence the development of NCDs such as type 2 diabetes, metabolic syndrome, and cardiovascular diseases (CVD). Hyperglycaemia is a common symptom in diabetic or obese patients and can drive maladaptive TI. Edgar et al., 2021 demonstrated that a ‘hyperglycaemic memory’ can be transmitted via transplantation of bone marrow from streptozotocin-induced diabetic mice into normoglycaemic *Ldlr^-/-^* mice, leading to an increased rate of atherosclerosis in recipient mice compared to controls. Epigenetic changes in HSCs were dependent on Runt-related transcription factor 1 (*Runx1*) and persisted even in prolonged culture of macrophages with physiological glucose levels. Similarly, pro-inflammatory epigenetic signatures were detected in monocytes of diabetic patients, which persisted for more than 6 years even when the patient’s glucose level normalised due to effective treatments (Miao et al., 2014).

Vinci et al., 2024 showed that under hyperglycaemic conditions, HSPCs undergo epigenetic changes including chromatin modifications leading to a senescence-associated secretory phenotype. This is marked by increased secretion of IL-6 and TNF and HSPCs mainly differentiated into pro-inflammatory monocytes (CD14^hi^CD16^+^). Furthermore, hyperglycaemia trains neutrophils to enhance neutrophil extracellular trap (NET) formation and release by increasing acetyl-CoA levels and promoting histone acetylation at histones 3 and 4 (H3K9ac, H3K14ac, H3K27ac, and H4K8ac). This heightened NET formation impairs wound healing by exacerbating inflammation in diabetic patients (Shrestha et al., 2024).

Additionally, hyperlipidaemia has been linked to maladaptive TI and the adipose tissue has been implicated in sustaining TI. Mice switched from a high-fat diet to a chow diet retain elevated adipose tissue inflammation, insulin resistance, and glucose intolerance, indicating long-lasting immune memory (Blaszczak et al., 2020).

Seufert et al., 2022 demonstrated that a ketogenic diet enriched in saturated fatty acids led to enhanced myelopoiesis in the bone marrow and an elevated inflammatory response to a systemic LPS stimulus. Similarly, free palmitic acid increased inflammation in vitro and mortality in vivo in response to a secondary stimulation with LPS several days later, implicating a role of palmitic acid in TI and worsening of metabolic diseases like metabolic syndrome. Furthermore, the adipocyte hormone leptin causes innate immune training by an enhanced TNF response one week later upon a secondary stimulus (Flores Gomez et al., 2024). Previously, statins were shown to prevent ox-LDL and β-glucan-induced TI in vitro. However, in patients with familial hypercholesterolaemia, the training phenotype persisted despite three months of statin therapy, as confirmed by RNAseq and CHIP-qPCR analysis (Bekkering et al., 2019). This result underlines the need for effective therapeutic approaches to tackle maladaptive TI in humans.

### Cardiovascular disease

CVDs are the leading cause of death in western countries, where vaccines and antibiotics have largely eliminated the threat of infectious diseases (van den Brink et al., 2019; Groh et al., 2018). CVDs affect the heart and blood vessels and are caused by lipid deposits and blood clots. Atherosclerosis is a dyslipidaemic state driven by low-grade chronic inflammation that favours the deposition of cholesterol in arterial walls, leading to atherosclerotic plaques (Libby, 2021). Atherosclerosis is initiated by myeloid cell infiltration into the intimal layer of the arterial wall and the formation of foam cells following the uptake of lipids. More immune cells are recruited to this inflammatory milieu, and the chronic inflammatory loop is sustained (Libby, 2021).

Trained monocytes have been isolated from patients with atherosclerosis and dyslipoproteinaemia (Bekkering et al., 2020; Riksen et al., 2023) and maladaptive TI may serve as a connection between infections and an elevated risk of CVD (Leentjens et al., 2018). Infections such as *Chlamydia pneumoniae*, cytomegalovirus and HIV are associated with an increased risk for atherosclerotic CVD (Pothineni et al., 2017).

CVD itself predisposes to comorbidities through maladaptive TI. Simats et al., 2024 transplanted bone marrow from mice subjected to an experimental model of ischaemic stroke by transient middle cerebral artery occlusion to recipient mice. The recipient mice had more CCR2^+^ monocytes invading cardiac tissues, developed cardiac fibrosis and cardiac dysfunction in comparison to control mice. Crucially, blocking IL-1β early after stroke (<1 hr) prevented the induction of both post-stroke myelopoiesis and the epigenetic training signature in the bone marrow, reducing cardiac inflammation and fibrosis. Moreover, inhibiting migration of trained myeloid cells by antagonising CCR-2 and CCR-5 with cenicriviroc was also cardioprotective (Simats et al., 2024).

Furthermore, myocardial infarction (MI) accelerates atherosclerosis in mice via increased bone marrow mobilisation and extramedullary monocytopoiesis mediated by sympathetic nervous signalling (Dutta et al., 2012). Adoptive bone marrow transfer in mice revealed that this effect is transmissible via HSPCs, and it was further shown that MI triggers a state of TI in monocytes. The acceleration of disease post-MI is not only restricted to atherosclerosis, as it has been shown that MI also elevates the risk for breast cancer in mice and humans (Koelwyn et al., 2020).

Mice receiving bone marrow from mice subjected to experimental heart failure by transverse aortic constriction developed spontaneous cardiac dysfunction and fibrosis and had increased vulnerability to kidney and skeletal muscle insults. Mechanistically, it was proposed that a sustained sympathetic nerve neuropathy in the bone marrow drives HSC proliferation due to impaired production of active transforming growth factor beta (TGF-β) (Nakayama et al., 2024). Inhibition of TGF-β activation reduces the ability of HSCs to return into a quiescent state, leading to enhanced haematopoiesis (Yamazaki et al., 2024).

In the context of haematopoiesis and maladaptive TI, clonal haematopoiesis of indeterminate potential (CHIP) is strongly age-associated. CHIP refers to the presence of white blood cells derived from a single dominant HSC clone that carries cancer-associated somatic mutations mostly occurring in genes encoding epigenetic regulators, specifically *TET2*, *DNMT3a*, and *ASXL1* (Chavakis et al., 2022). Clonal haematopoiesis and CHIP increase the risk for cardiovascular events, and CHIP in particular is a predictor for adverse outcomes in patients with atherosclerosis (Dregoesc et al., 2024; Gumuser et al., 2023). Murine studies have shown that *Tet2* and *Dnmt3* mutations are associated with HSPC expansion and a bias towards myeloid lineages including an increased pro-inflammatory potential that fuels into disease progression (Fuster et al., 2017; Rauch et al., 2023). Increased bone marrow IL-1β levels during ageing drive *Tet2^+/−^* clonal haematopoiesis and this effect was abolished upon genetic deletion of *Il1r1* in mice, exemplifying the complexity of intertwining pathologies via IL-1 signalling (Caiado et al., 2023). In other words: having different causes, both CHIP and central TI are characterised by HSPCs with enhanced myelopoiesis and hyper-responsive progeny. For further details about CHIP, atherosclerosis and maladaptive TI, we refer to other reviews (Chavakis et al., 2022; Hajishengallis et al., 2022).

Reversing or preventing TI in CVD might provide new therapeutic options for treating patients with chronic metabolic diseases such as obesity, diabetes mellitus and metabolic syndrome to decrease the risk of CVD as a secondary burden (Charles‐Messance and Sheedy, 2021). For mechanistic insights into TI cardiometabolic diseases, we refer to recent reviews (Riksen et al., 2023; Bahrar et al., 2024).

### Gout

Gout is characterised by a state of hyperuricaemia followed by the deposition of inflammatory monosodium urate (MSU) crystals in joints, cartilage and connective tissue (ChenXu et al., 2019). Hyperuricaemia may arise from loss-of-function mutations in enzymes involved in purine metabolism or urate transporters. Additionally, risk factors for hyperuricaemia include lifestyle and dietary factors, such as a high purine diet, obesity, and dehydration. Gout flares occur when MSU crystals activate the NLRP3 inflammasome and bioactive IL-1β is released. Urate itself is thought to be a key factor in the persistence and recurrence of inflammation in gout patients, beyond contributing to crystal deposition (Cabău et al., 2020). Indeed, urate stimulation of PBMCs and monocytes ex vivo from gout patients induces a TI phenotype, characterised by a heightened pro-inflammatory cytokine response to secondary stimuli compared to healthy controls. This TI response could be abolished with pharmacological inhibition of histone methylation, highlighting the role of epigenetic modifications, another hallmark of TI, in gout pathology (Crisan et al., 2016; Crisan et al., 2017). Another significant aspect of gout flares is the infiltration of neutrophils into the synovial tissue and fluid, where serine proteases and other pro-inflammatory mediators are released. Neutrophils, despite being short-lived and terminally differentiated cells, have been shown to undergo central TI at the progenitor cell level, resulting in trained neutrophils (Moorlag et al., 2020). Of note, gout flares result from recurrent heightened inflammatory responses that persist long after initial crystal deposition. This hyper-responsiveness alongside epigenetic remodelling is characteristic of TI and not transient or chronic priming.

**Figure 1.**
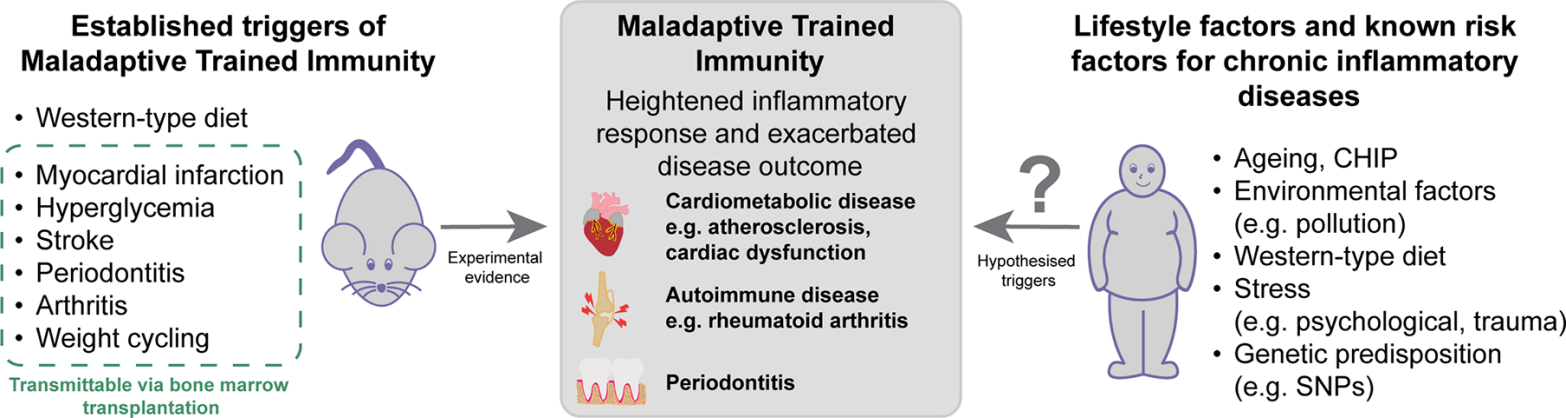
Maladaptive trained immunity (TI) leads to a heightened inflammatory response and exacerbated disease outcomes. Using murine models, different triggers have been identified, and studies using bone marrow transplantation show that central reprogramming within the bone marrow is pivotal for maladaptive TI. These experiments demonstrate that long-lasting epigenetic and metabolic changes in HSPCs drive persistently heightened inflammatory responses and as such contribute to chronic, dysregulated inflammation. In humans, evidence remains scarcer, yet multiple lifestyle factors, as well as established risk factors for chronic inflammatory diseases, have been proposed as potential triggers of maladaptive TI. These factors are known to enhance systemic inflammation and may similarly imprint long-term changes on immune cells.

Taken together, TI in gout may amplify the immune response beyond initial crystal deposition and contributes to the persistence and recurrence of inflammation, exacerbating disease severity. Over successive flares, this heightened inflammatory state of maladaptive TI could lead to an increased risk of chronic arthritis and high incidence of comorbidities, in response to subsequent stimuli. Similarly, TI may increase susceptibility to gout flares resulting in an ongoing inflammatory feedback loop. Yet, further evidence is needed to highlight the role of urate-induced metabolic reprogramming in the context of maladaptive TI (Figure 1).

## Lifestyle modulation as a strategy to prevent or reverse maladaptive TI

Lifestyle changes such as improving dietary habits and physical activity can modulate or even erase pre-existing TI (Table 1). For instance, dietary adjustments, weight management and alcohol moderation benefit gout patients by alleviating symptoms, reducing uric acid and thus the likelihood of crystal formation, preventing TI induction and limiting disease progression (Zhang et al., 2022). Similarly, hypocaloric diets and fasting reduce systemic inflammation in patients with metabolic, autoimmune, and inflammatory diseases (Gopalarathinam et al., 2024; Gasmi et al., 2018). Moreover, Jordan et al., 2019 fasted mice for 19 hours and observed increased activation of the low-energy sensor 5′-AMP-activated protein kinase (AMPK) in hepatocytes and reduced systemic CCL2 production, and reduced mobilisation of monocytes into circulation. Any accompanying weight loss should be maintained to prevent ‘weight cycling’, which has been shown to increase the risk for atherosclerosis in mice due to neutrophil reprogramming in the bone marrow (Lavillegrand et al., 2024). Adipose tissue macrophages of weight-cycled mice showed higher basal levels of TNFα and increased basal and maximal respiration which indicate maladaptive TI (Caslin et al., 2023).

**Table 1.** Suggested lifestyle interventions to prevent or reduce maladaptive TI-related inflammation.

Lifestyle modulation	Disease type	Cellular and molecular targets	Possible off-target effects	References

Dietary and weight management, alcohol moderation	Gout	Reduction of uric acid prevents crystal formation in the joints	Nutrient deficiencies	Zhang et al., 2022
Hypocaloric diet, fasting	Metabolic, autoimmune, and inflammatory diseases	Reduction of systemic inflammation via activation of hepatic AMPK and reduction of systemic CCL2	Nutrient deficiencies	Gopalarathinam et al., 2024; Gasmi et al., 2018
Prevention of alternating periods of highly processed foods and ‘healthy’ food	Atherosclerosis	Prevention of IL1β-dependent neutrophil reprogramming in the BM	n/a	Lavillegrand et al., 2024
Prevention of weight cycling	Atherosclerosis	Prevention of HSPC reprogramming	n/a	Scolaro et al., 2025
Moderate exercise	Cardiovascular disease	Metabolic rewiring of macrophages: increased OXPHOS and reduced ROS production	n/a	Murugathasan et al., 2023; Zaid et al., 2024

Moderate exercise reduced the inflammatory response after in vitro LPS stimulation of murine BMDMs when compared to inactive mice. These changes were induced by persistent metabolic rewiring and changes to chromatin accessibility that led to an increased expression of anti-inflammatory macrophage-related genes. Additionally, higher mitochondrial quality and higher reliance on mitochondrial OXPHOS, with reduced ROS production, was observed (Murugathasan et al., 2023). The effects of exercise on inflammation and cardiovascular risks have been recently summarised by Zaid et al., 2024. Exercise could be considered a therapeutic approach to either increase the threshold for TI induction or to reverse maladaptive training, but this requires further investigation.

Considering the above-mentioned links between CPS and TI, as well as its additional effect on other parameters, such as dietary choices and physical activity levels, treating and preventing CPS and increasing mental resilience could also be beneficial to prevent maladaptive TI. Among such interventions, one should consider measures that maintain a healthy level of oxytocin. Oxytocin, a key mediator of parturition, lactation, and social behaviour (Froemke and Young, 2021), has recently been linked mechanistically to cardiovascular health. Studies in mice have shown that social isolation reduced plasma levels of oxytocin and worsened atherosclerosis due to changes in hepatic lipid metabolism (Ko et al., 2025). Further, a proteome-wide association study of plasma proteins from the UK Biobank linked five proteins to loneliness and social isolation, which were further related to inflammation and to the development of CVD, depression, and type II diabetes (Shen et al., 2025). Further experiments could tackle the question whether immune cells from socially isolated individuals show a training phenotype. Finally, in our modern age, the growing use of social media and a drop in in-person communication have a high relevance (Schnepf et al., 2024). Structural changes in affected populations should be considered to ameliorate loneliness and increase well-being.

## Limitations and future directions

Various reports from the past decade indicate that maladaptive TI can contribute to the onset or exacerbation of chronic inflammatory diseases. Most in vivo mechanistic evidence still comes from mouse models, and most available human data reveal association and not causation (Figure 1).

The prevalence of NCDs, coinciding with increased maladaptive effects of TI, has risen globally over the last few decades during which human-made interventions both increased life expectancy and prolonged exposure to modern lifestyle-associated training stimuli. This is a negligible amount of time in comparison to the evolutionary timeline along which TI has been beneficial. Each individual may have an individual threshold and capacity for TI induction and retention. A clinical test for current training status, as well as trainability compared to baseline, would be a useful diagnostic tool to assess whether maladaptive TI contributes to disease and which patients may benefit from therapeutic intervention. Furthermore, clinical trials that assess lifestyle interventions, for example, Western diet versus fibre-rich diet (Temba et al., 2025) may provide a deeper understanding of maladaptive TI in humans, if TI metrics were assessed.

Despite growing recognition of the role of environmental- and lifestyle-associated influences on TI, the exact influence of many such factors remains largely unresolved. Chronic exposure to pollutants, sensory ecology factors, regional conditions, and endemic microbes may shape the immune system by modulating circadian rhythms, physiological and psychological stress responses, and baseline inflammatory set points. All of these influences may be further modified by cultural factors, including the prevalence of specific hygiene or medical practices, as well as individual- or community-level socio-economic context and associated differences in access to healthcare, education, adequate housing, and clean drinking water. Considering the emerging central role of the microbiome in both innate and adaptive immune regulation in mammals, it will be particularly important to understand how these factors, together with differences between rural and urban lifestyles, shaping the microbiome. Microbiome-dependent pathways may form a key integrative axis linking diverse exposures to trained immune states.

We consider that it is often very difficult to distinguish (chronic) priming from TI in a real-world or clinical setting, in which there is no sterile environment. A clear distinction would require an in-depth integrative analysis of immunological, epigenetic, metabolic, and functional profiling of individuals in a longitudinal fashion. As such, a dataset would need to contain information on a cell (or organism’s state) at (1) baseline, (2) after the primary stimulus, (3) whether it returns to baseline or not, and (4) its state after the secondary stimulus has been resolved.

We conclude that TI once served as an adaptive immune response in innate cells that greatly benefitted a species’ success in pathogen-rich environments. Yet, TI becomes maladaptive in the face of modern lifestyle exposures and an ageing population. TI has indeed become hijacked by poor diet, stress, and pollutants and may now underlie the chronic inflammation driving the current epidemic of NCDs.
